# Modulation of OMV Production by the Lysis Module of the DLP12 Defective Prophage of *Escherichia coli K12*

**DOI:** 10.3390/microorganisms9020369

**Published:** 2021-02-12

**Authors:** Martina Pasqua, Alessandro Zennaro, Rita Trirocco, Giulia Fanelli, Gioacchino Micheli, Milena Grossi, Bianca Colonna, Gianni Prosseda

**Affiliations:** 1 Istituto Pasteur Italia, Dipartimento di Biologia e Biotecnologie “C. Darwin”, Sapienza Università di Roma, Via dei Sardi 70, 00185 Rome, Italy; martina.pasqua@uniroma1.it (M.P.); alessandro.zennaro@uniroma1.it (A.Z.); rita.trirocco@uniroma1.it (R.T.); giulia.fanelli@uniroma1.it (G.F.); milena.grossi@uniroma1.it (M.G.); bianca.colonna@uniroma1.it (B.C.); 2 Istituto di Biologia e Patologia Molecolari, Consiglio Nazionale delle Ricerche (CNR), P. le Aldo Moro 5, 00185 Roma, Italy; gioacchino.micheli@uniroma1.it

**Keywords:** OMV, cryptic prophage, lysis module, cell wall, phage domestication

## Abstract

Outer membrane vesicles (OMVs) are nanostructures mostly produced by blebbing of the outer membrane in Gram negative bacteria. They contain biologically active proteins and perform a variety of processes. OMV production is also a typical response to events inducing stress in the bacterial envelope. In these cases, hypervesiculation is regarded as a strategy to avoid the dangerous accumulation of undesired products within the periplasm. Several housekeeping genes influence the biogenesis of OMVs, including those correlated with peptidoglycan and cell wall dynamics. In this work, we have investigated the relationship between OMV production and the lysis module of the *E. coli* DLP12 cryptic prophage. This module is an operon encoding a holin, an endolysin and two spannins, and is known to be involved in cell wall maintenance. We find that deleting the lysis module increases OMV production, suggesting that during evolution this operon has been domesticated to regulate vesiculation, likely through the elimination of non-recyclable peptidoglycan fragments. We also show that the expression of the lysis module is negatively regulated by environmental stress stimuli as high osmolarity, low pH and low temperature. Our data further highlight how defective prophages finely contribute to bacterial host fitness.

## 1. Introduction

Outer Membrane Vesicles (OMVs) are spherical, double-layered membrane nanostructures ranging in size from 20 to 250 nm derived from the cell envelope of Gram negative bacteria [1,2]. They originate mainly from the blebbing of the outer membrane and are composed by an outer leaflet of lipopolysaccharide and an inner leaflet of phospholipids [3,4,5]. Besides being enriched in outer membrane proteins, OMVs contain bioactive components, including periplasmic and cytoplasmic proteins, peptidoglycan, lipopolysaccharides (LPS), DNA, RNA and enzymes [3,6].

OMVs have been correlated with different functions, including cellular detoxification, intercellular communication, release of virulence factors, DNA transfer, resistance to bacteriophages, antibiotics and host defence factors [7,8,9,10,11,12]. Accordingly, OMVs are regarded as an additional strategy adopted by bacteria to increase their chances of survival and to optimize their adaptation to particular niches.

Vesiculation levels can be modulated by several environmental conditions such as temperature, nutrient availability, growth conditions, quorum sensing and envelope targeting antibiotics [3]. Growth conditions have significant influence on the vesiculation process and cells in stationary growth phase show maximum OMV yields. OMV production is also a response to envelope stress caused by the accumulation of different products in the envelope. Indeed, the alteration of the peptidoglycan (PG) and of the bonds between the cell wall and LPS layer, as well as the accumulation of PG fragments, intermediates of LPS synthesis and misfolded proteins have been correlated to higher production of OMVs [3,13,14]. These observations are supported by the identification, in several bacterial species, of genes whose inactivation increases vesiculation. In most cases, these genes encode functions that are useful either for envelope biosynthesis or to avoid accumulation of unwanted products in the periplasm [4]. For example, loss of DegP, a periplasmic protease/chaperone involved in the control of envelope stress caused by misfolded proteins, leads to hypervesiculation [15]. Similarly, accumulation of PG fragments in strains defective in the *ampG* and *amiD* genes, encoding an inner membrane permease and an amidase, causes an increase of OMV production [14]. Hypervesiculation has also been observed in strains harboring mutations (e.g., in the *rfaC* and *rfaG* genes) that alter the sugar core of the LPSs, leading to accumulation of LPS fragments [14,16].

Recently, we have shown that the introduction of a functional lysis module of the *Escherichia coli* cryptic prophage DLP12 into *Shigella flexneri* induces an increased secretion of peptidoglycan fragments from the cell wall [17]. DLP12 is a defective lambdoid prophage located at 12 min on the *E. coli* genome [18]. This cryptic prophage has conserved a large part of its genome, including the lysis module which is known to contribute to cell wall maintenance and biofilm formation [19]. *S. flexneri* shares a high genome homology with the commensal *E. coli* and on the basis of phylogenetic analyses has been included within the *E. coli* species [20,21]. Several studies have shown that during the evolution towards pathogenicity *Shigella* has lost several genes present on the *E. coli* chromosome which would have been deleterious for the full expression of virulence and for the interaction with the host [22,23,24]. Among the lost genes, there is the locus containing the lysis module of DLP12 phage (Dlm). Indeed, restoring Dlm functionality in *Shigella* increases the release of peptidoglycan fragments, which over-stimulate the host innate immunity, ultimately leading to virulence mitigation [17].

The Dlm consists of a single operon containing four genes, *essD*, *ybcS* and *rzpD*/*rzoD* [19], which are homologs of the lambda phage genes encoding the cell-lysis proteins S (holin), R (endolysin) and R_z_/R_z1_ (spannins) [25]. Holin forms small pores through which endolysin and spannins reach the periplasmic space where they cooperate, causing bacterial lysis [26]. The expression of the SRR_z_/R_z1_ operon is submitted to the control of several factors, including the phage Q type antiterminator [27], the host sigma factor RpoE [28] and the transcriptional regulator Hha [29].

Considering the involvement of the SRR_z_/R_z1_ operon in envelope maintenance and the relationship between OMV biogenesis and cell envelope stability, we asked whether OMV production is affected by the expression of the SRR_z_/R_z1_ genes. The data we present indicate that the absence of the DLP12 SRR_z_/R_z1_ operon in *E. coli* strongly increases OMV production. In addition, we show that this increase depends on the loss of the entire operon whereas the inactivation of single genes does not induce hypervesiculation. Finally, we show that the expression of the SRR_z_/R_z1_ operon is responsive to environmental stimuli such as osmolarity, pH and temperature.

## 2. Materials and Methods

### 2.1. Bacterial Strains and General Growth Conditions

The bacterial strains used in this study are listed in Table 1. ULS153 is a MG1655 derivative lacking the entire *lac* operon [30]. ULS153 derivatives were obtained using the one-step method of gene inactivation with pKD3 as template [31]. ULS153 derivative carrying a deletion of the DLP12 lysis module (ΔDlm) was obtained using the oligo pair datDlmF/datDlmR. The deletion mutants *essD* (ΔS), Δ*ybcS* (ΔR) and Δ*rzpD*/*rzoD* (ΔRz) were obtained using the oligo pairs, datSF/datSR, datRF/datRR and datRzF/datRzR, respectively (Table S1). To avoid polar effects, the Cm^R^ gene cassette was eliminated exploiting the flippase encoded by the pCP20 plasmid using flippase/flippase recognition target (Flp/FRT) recombination [31]. Following the flippase procedure, double mutants were obtained from single mutant strains using the one-step method mentioned above. ULS153 ΔSR and ΔSRz double mutants were obtained by inactivating the *ybcS* or *rzpD*/*rzoD* genes of strain ULS153 ΔS. The ULS153 ΔRRz double mutant was obtained by inactivating the *rzpD*/*rzoD* gene of the ULS153 ΔR strain. Gene deletions in double mutant strains were obtained using the same oligo pairs listed in Table S1. The ULS153 derivative lacking the *degP* gene was obtained by P1 transduction using the JW0157-1 ∆*degP* strain from the Keio Collection [32] as a donor. Cells were routinely grown aerobically in Luria-Bertani (LB) medium at 37 °C. When required, bacteria were grown in M9 minimal medium supplemented with 10 µg/mL thiamine, 0.5% casamino acids, 1 mM MgSO_4_, 0.1 mM CaCl_2_ and 0.2% glucose as carbon sources (M9 complete medium). To test the effect of osmolarity, NaCl (0.15 and 0.3 M) or sucrose (0.3 and 0.6 M) were added to M9 complete medium. To test the effect of pH, the M9 complete medium was adjusted to the desired value by altering the Na_2_HPO_4_ and KH_2_PO_4_ relative concentrations. Solid media contained 1.6% agar. Antibiotics were used at the following concentrations: ampicillin 100 µg/mL; chloramphenicol 25 µg/mL; kanamycin 30 µg/mL; streptomycin 10 µg/mL.

### 2.2. Plasmid Construction

The plasmids used in this study are listed in Table 1. pAC2.10 is a pACYC184 derivative containing the DLP12 lytic module from MG1655 [17]. To assay the activity of the SRR_z_/R_z1_ promoter, the regulatory region of the operon was cloned upstream the promoter-less *lux* reporter operon carried by the pAG4 vector [33]. To this end, an amplicon comprising the transcriptional regulatory region of the SRR_z_/R_z1_ operon and ranging from −121 to +650 [28] was obtained using the oligo pair Pdlp12Fw/Pdlp12Rv and MG1655 as template and first cloned into the pGEM-T easy vector and then transferred into the *PstI* site of pAG4. The same strategy was used to construct pAGP*lac*. In this case, the PCR product was obtained using the oligos PlacKD13F and PlacKD13R and total DNA of MG1655 as a template and the amplicon covers the −56 to +1 region considering the +1 P*lac* transcriptional starting site.

### 2.3. General Procedures

DH10b was used as recipient strain in cloning procedures. Plasmid DNA extraction, DNA transformation, cloning, restriction, electrophoresis and purification of DNA fragments were performed as previously described [34,35]. PCR reactions were routinely performed using DreamTaq DNA polymerase (Thermo Fischer Scientific, Waltham, MA, USA). Ex Taq DNA polymerase (TaKaRa, Shiga, Japan) was adopted to obtain longer transcripts and high fidelity. All oligonucleotides used in this study are listed in Table S1 and have been designed mainly on the basis of the genomic sequence of *E. coli* K-12 MG1655. Deletion mutants and plasmid constructs were verified by DNA sequencing performed by BioFab, Rome, Italy.

### 2.4. Biosensor Assay

*E. coli* ULS153 harboring pAGPDlm, pAGP*lac* or pAG4 (as negative controls) were used to assay luminescence expression. Bacteria were grown in M9 complete medium under different environmental conditions. Then, 200 μL of each sample were transferred to a 96-well microtiter plates and the O.D._600_ and luminescence were rapidly measured. Luminescence was measured at 485-nm excitation and 535-nm emission wavelengths with a CLARIOstar plate reader (BMG LABTECH, Offenburg, Germany). The reporter activity was determined as relative luminescence units (RLU) normalized to the OD_600_ of each sample in order to account for bacterial cell concentration. The ULS153 pAG4 reporter activity was subtracted from each measured sample, the resulting values were normalized to the ULS153 pAGP*lac* reporter activity obtained under the same conditions and finally the obtained values were compared with the chosen reference sample set to one.

### 2.5. OMV Purification

OMVs were isolated from culture supernatants according to previous studies with slight modifications [36]. Briefly, bacterial cells from overnight cultures were sub-cultured in 500-mL flasks containing 50 mL of LB broth at 37 °C with vigorous shaking (200 rpm). After 15 h of growth, bacterial cultures were pelleted by centrifugation at 6000 rpm for 15 min at 4 °C. Supernatants were collected for OMV purification, while bacterial pellet were used for total protein extraction. The supernatants were filtered (0.45 μm pore size) to remove residual bacteria and concentrated by centrifugation in a 100 K Amicon^®^ Ultra-15 centrifugal filter device (Merck Millipore, Tullagreen, Carrigtwohill, Ireland) at 5000 rpm for 15 min at room temperature using a swing-out rotor. OMVs were washed by adding 15 mL of PBS to the centrifugal filter device and repeating the centrifuge step. Finally, OMVs were recovered by inverting the filter device and centrifuging as described in the manufacturer’s instructions. PBS 1× was added to samples, bringing the volume to 60 μL.

### 2.6. SDS-PAGE and Immunoblot Analysis

Total protein extracts from purified OMVs were prepared as described above. 20 μL aliquots were mixed with Laemmli SDS sample buffer (Alfa Aesar, Thermo Fisher Scientific, Waltham, MA, USA), boiled at 100 °C for 5 min and run on 10% SDS-PAGE in parallel with molecular weight markers (Page Ruler; Thermo Fisher, Waltham, MA, USA). Gels were stained with Coomassie Blue (0.1%). OMV production was quantified on the basis of OmpF/C band using the densitometry tool of the Image Lab software (Bio-Rad, Hercules, CA, USA), normalizing against the wt strain.

For immunoblot analysis, SDS-PAGE profiles were transferred to PVDF membranes (Hybond-P, Millipore, Tullagreen, Carrigtwohill, Ireland). The membranes were incubated overnight at 4 °C in a 1:50,000 dilution of a *E. coli*-OmpA rabbit antibody according to Ambrosi et al. [37], while the anti-rabbit secondary antibody was used in a 1:10,000 dilution. The protein-antibody complex was detected by enhanced chemiluminescence (Euroclone, Pero, Mi, Italy), as previously described [38]. The same densitometry procedure was followed to estimate OmpA abundance in the mutant strains, normalizing against the wt strain.

### 2.7. DiO Staining

A fluorescence assay with Vybrant™ DiO Cell-Labeling Solution (Invitrogen; Thermo Fisher Scientific, Waltham, MA, USA) was performed in order to confirm OMV quantification of ULS153 and ULS153 ΔDlm strains. For each sample, 15 µL of purified OMVs were suspended in 85 µL of PBS and the mixture was labeled with 1% Vybrant™ DiO by incubation for 20 min at 37 °C [39]. Free dye was removed by two washes with 15 mL of PBS using the 100K Amicon^®^ Ultra-15 centrifugal filter device. Labeled OMVs were collected and stored at −20 °C. The concentration of OMVs was determined by measuring fluorescence at 485-nm excitation and 535-nm emission wavelengths with a Wallac 1420 Victor^3^V microplate reader (Perkin-Elmer, Waltham, MA, USA).

### 2.8. Statistical Analyses

The statistical significance of data was determined using Excel, Microsoft 365 by calculating the *p* values derived from a two-tailed *t*-test.

**Table 1:** Strains and Plasmids.

*E. coli* Strain	Relevant Characteristics	Source/Reference
DH10b	*E. coli* K12	[40]
MG1655	*E. coli* K12, F^-^, λ^-^, *ilvG*^-^, *rfb-50*, *rph-1*	[41]
ULS153	ΔlacZYA derivative of MG1655	[30]
ULS153 ΔD**lm**	ULS153 mutant defective in DLP12 operon	This study
ULS153 Δ*degP*	ULS153 mutant defective in *degP* gene	This study
ULS153 ΔS	ULS153 mutant defective in *essD* gene	This study
ULS153 ΔR	ULS153 mutant defective in *ybcS* gene	This study
ULS153 ΔRz	ULS153 mutant defective in *rzpD*/*rzoD* genes	This study
ULS153 ΔSR	ULS153 mutant defective in *essD* and *ybcS* genes	This study
ULS153 ΔRRz	ULS153 mutant defective in *ybcS* and *rzpD*/*rzoD* genes	This study
ULS153 ΔSRz	ULS153 mutant defective in *essD* and *rzpD*/*rzoD* genes	This study
**Plasmid Name**	**Relevant Characteristics**	**Source/Reference**
pACYC184	Low/Medium copy number cloning vector	[40]
pAC2.10	pACYC184 derivate carrying the DLP12 operon	This study
pKD46	Red recombinase expression plasmid	[31]
pKD3	Template plasmid carrying a Cm^R^ gene with FLP recognition target sequence	[31]
pCP20	Temperature sensitive replicon carrying the yeast Flp recombinase gene	[31]
pAG4	High copy number plasmid carrying the lux operon	[33]
pAGPDlm	pAG4 derivate carrying the DLP12 promoter region of MG1655 strain upstream lux operon	This study
pAGP*lac*	pAG4 derivate carrying the lac promoter region of MG1655 strain upstream lux operon	This study

## 3. Results

### 3.1. The SRR_z_/R_z1_ Operon Regulates OMV Production

**Figure 1:**
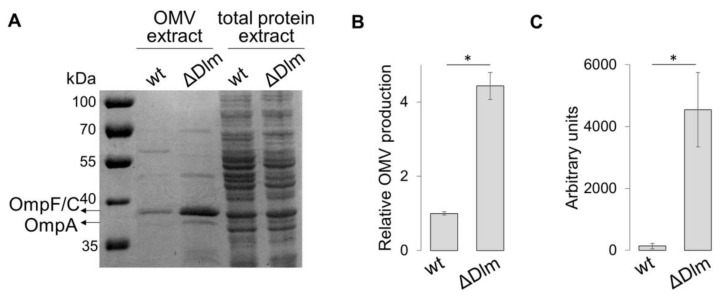
Deletion of the DLP12 lytic module correlates with an increased outer membrane vesicle (OMV) production (**A**) SDS-PAGE separation of OMV proteins and total proteins from ULS153 (wt) and its derivative lacking the DLP12 lysis module (ΔDlm). The position of the OmpF/C and OmpA proteins is indicated by the arrows. (**B**) OMV production by ULS153 ΔDlm normalized to that by the wt. OMV quantification was obtained by densitometry of the OmpF/C band in SDS-PAGE separations. (**C**) DiO fluorescence measurement of OMV abundance in ULS153 ΔDlm relative to the wt. Vertical bars in B and C indicate the standard deviation of three independent experiments. Statistical significance was determined by the *t* test, and *p* values are as follows: * *p*  <  0.01.

Accumulation of cell envelope components or misfolded proteins in the periplasmatic space leads to an hypervesiculation phenotype [13,14]. Since the products of the DLP12 lysis module (Dlm) contribute to the release of non-recyclable PG fragments [17,19], we decided to evaluate the involvement of the Dlm operon in modulating vesiculation. The protein profiles of ultrafiltration-purified OMVs from *E. coli* strain ULS153 (wt) and its isogenic derivative ULS153 ∆Dlm, which harbors a deletion of the entire SRR_z_/R_z1_ operon (Table 1), were compared by using SDS-PAGE. The OmpF and OmpC porins (OmpF/C) were used as relative quantitative markers, since these membrane proteins are expressed specifically and abundantly within the outer membrane and therefore predominate in OMVs [13,36]. Figure 1A,B show an about 5-fold increase of the OmpF/C protein population in the OMV preparation from ULS153 ∆Dlm as compared to that from the wt, while total protein extracts from equal density cultures are closely comparable in both strains. This result has been confirmed by a fluorescent assay with Vybrant DiO, a lipophilic membrane stain that diffuses laterally in membranes and that is weakly fluorescent until incorporated into membranes [39]. As shown in Figure 1C, fluorescence from the ULS153 ∆Dlm OMV preparation is highly increased as compared to the wt, indicating that the loss of the DLP12 lysis module gives rise to an intense production of OMVs.

**Figure 2:**
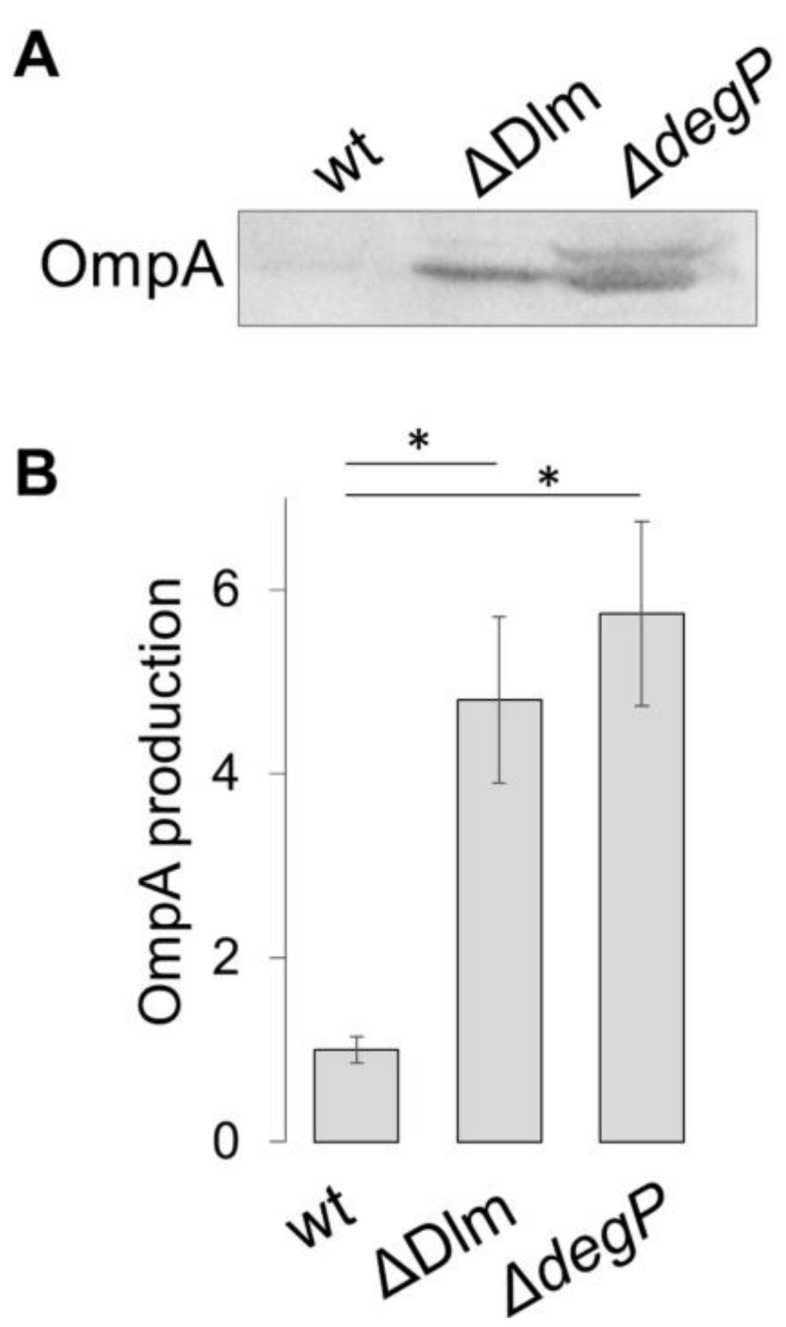
Comparative analysis of OMV production in ΔDlm and Δ*degP* background. (**A**) Western blot analysis of OmpA in OMVs isolated from ULS153 (wt), ULS153 ΔDlm and ULS153 Δ*degP*. Blots of SDS-PAGE separations were probed with anti-*E. coli* OmpA antibody. (**B**) Relative OmpA abundance determined by densitometry. The OmpA bands of the ΔDlm and Δ*degP* strains were normalized to that of the wt. Vertical bars indicate the standard deviation of three independent experiments. Statistical significance was determined by the *t* test, and *p* values are as follows: * *p*  <  0.01.

To better evaluate the level of OMV production in the ΔDlm mutant with respect to a background well known to induce hypervesiculation, we constructed a ULS153 derivative carrying a *degP* deletion (ULS153 Δ*degP*). DegP is a periplasmic protease/chaperone that manages unfolded and misfolded proteins [42]. In *E. coli degP* mutants accumulate misfolded envelope proteins in the periplasm, giving rise to increased OMV production [15]. On this basis, electrophoretic OMV profiles from strains ULS153 (wt), ULS153 Δ*degP* and ULS153 ΔDlm were probed by Western blot with antibodies against OmpA, one of most abundant *E. coli* outer membrane proteins, used similarly to OmpF/C as specific marker for OMV quantitation [13,36]. The results (Figure 2) reveal that in the ΔDlm background OMV production is greatly increased (about 4.8-fold) as compared to the wt, closely approaching the level observed in the *degP* mutant (about 5.6 fold), thus, confirming the involvement of the Dlm sequence in influencing OMV production. The increase we observed in the *degP* mutant is perfectly in line with previous estimates in the same background [43].

**Figure 3:**
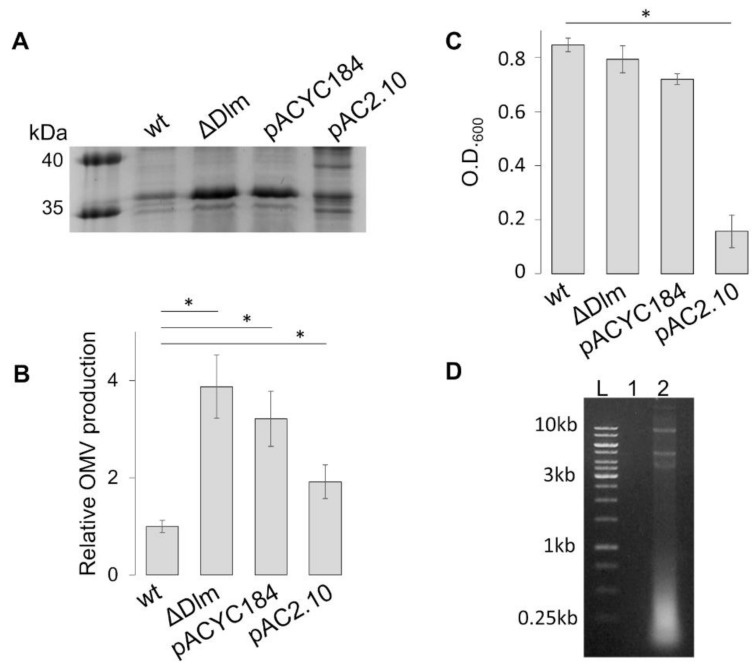
Trans-complementation of the SRRz/Rz1 operon confers a lytic phenotype to ULS153 ∆Dlm. (**A**) SDS-PAGE separation of OMV proteins from ULS153 (wt), ULS153 ΔDlm), UL153 ∆Dlm carrying the pACY184 vector (∆Dlm pACY184), and UL153 ∆Dlm carrying pAC2.10, a pACYC184-derived plasmid harboring a functional copy of the SRRz/Rz1 operon (∆Dlm pAC2.10). (**B**) OMV production of ULS153 ΔDlm, ULS153 ∆Dlm pACY184 and ULS153 ∆Dlm pAC2.10 normalized to the ULS153 wt strain. OMV amount was obtained by densitometry of the OmpF/C band in SDS-PAGE separations. (**C**) Growth measurements (OD_600_) of ULS153 wt, ULS153 ΔDlm, ULS153 ΔDlm pAC184 and ULS153 ΔDlm pAC2.10 after overnight cultures. Vertical bars indicate the standard deviation of three independent experiments. Statistical significance was determined by the *t* test, and *p* values are as follows: * *p*  <  0.01. (**D**) Agarose gel separation of DNA purified from overnight culture supernatants of ULS153 ∆Dlm pACYC184 (lane 1) and ∆Dlm pAC2.10 (lane 2). The presence of DNA is detectable only in ULS153 ∆Dlm pAC2.10. L, mw markers (kb).

Next, we tested whether the overproduction of OMVs in the ΔDlm background could be complemented by the expression of the deleted genes on a plasmid vector. To this end, we transformed ULS153 ∆Dlm with pAC2.10, a pACYC184-derived plasmid harboring a functional copy of the SRR_z_/R_z1_ operon [17]. The comparison of OMV protein profiles (Figure 3A,B) indicates that the presence of pAC2.10 in the ∆Dlm background only partially restores the profile of the wt strain (ULS153). The plasmid vector is not involved, since the protein profiles of ULS153 ∆Dlm and ULS153 ∆Dlm pACYC184 are comparable. To explain the partial complementation, we asked whether an increased expression of the SRR_z_/R_z1_ operon carried by pAC2.10 might be toxic for the cells. To test this hypothesis, we first checked the OD_600_ value reached after overnight growth by strains ULS153, ULS153 ∆Dlm, ULS153 ∆Dlm pACYC184 and ULS153 ∆Dlm pAC2.10. As shown in Figure 3C, the ectopic presence of the SRR_z_/R_z1_ operon (ULS153 ∆Dlm pAC2.10) causes a substantial decrease (about 5-fold) of the OD_600_ as compared to the other strains, suggesting a lytic effect due to overexpression of the DLP12 lysis module. Cell lysis was further confirmed by the abundant presence of nucleic acids in the culture supernatants from ULS153 ∆Dlm pAC2.10 and by their absence in supernatants from ULS153 ∆Dlm pACYC184 (Figure 3D). Altogether, these results indicate that an increased expression of the SRR_z_/R_z1_ operon confers a lytic phenotype to the ULS153 ∆Dlm strain.

### 3.2. Defining the Role of the Single Genes of the SRR_z_/R_z1_ Operon

**Figure 4:**
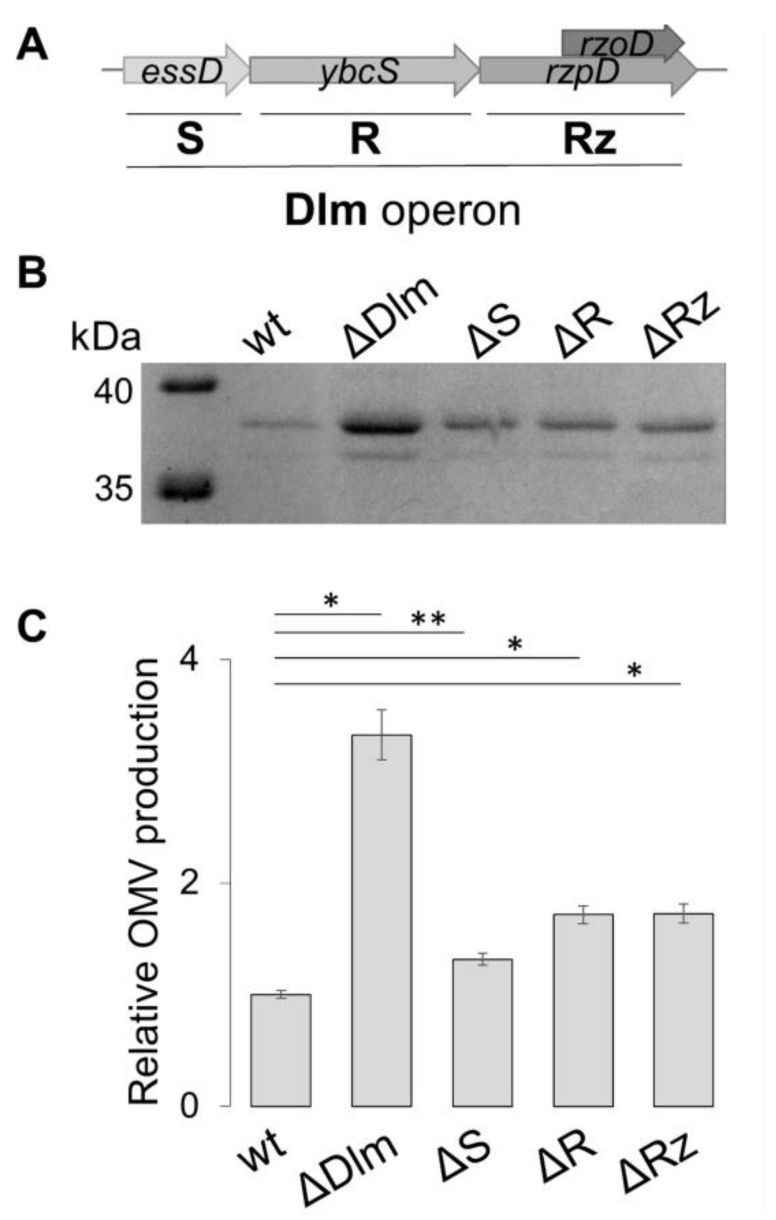
OMV production in ULS153 derivative strains lacking single genes of the DLP12 lysis module. (**A**) Schematic representation of the SRRz/Rz1 (Dlm) operon. Four genes are present: *essD*, encoding the holin (S); *ybcS* encoding endolysin (R); and *rzpD/rzoD* encoding the spannins (Rz). (**B**) SDS-PAGE separation of OMV proteins from ULS153 (wt) and its derivatives lacking the entire DLP12 lytic module (ΔDlm), the *essD* gene (ΔS), the *ybcS* gene (ΔR), or the *rzpD*/*rzoD* genes (ΔRz). (**C**) OMV production by ULS153 ΔDlm, ΔS, ΔR or ΔRz normalized to the wt. OMV amount was obtained by densitometry of the OmpF/C band in SDS-PAGE separations. Vertical bars indicate the standard deviation of three independent experiments. Statistical significance was determined by the *t* test, and *p* values are as follows: * *p*  <  0.01, ** *p* < 0.05.

Does the production of OMVs depend on the absence of single genes of the SRR_z_/R_z1_ operon? To answer this question, we used a site-specific mutagenesis approach to construct ULS153 derivatives defective for *essD*, *ybcS* or *rzpD*/*rzoD* genes (Figure 4A). The protein profiles associated with the OMV fraction of ULS153 Δ*essD*, ULS153 Δ*ybcS* and ULS153 Δ*rzpD*/*rzoD* were analysed by SDS-PAGE (Figure 4B). The OmpF/C fraction of the Δ*essD*, Δ*ybcS* and Δ*rzpD*/*rzoD* ULS153 derivatives is slightly enriched as compared to the wild type (ranging from 1.3-fold in the Δ*essD* mutant to 1.7-fold for the other mutants), but none of the mutants attains the high OmpF/C level observed in the ULS153 ΔDlm strain lacking the entire lysis module (Figure 4C). This indicates that the lack of functionality of individual genes of the lysis module is not sufficient to elicit full OMV overproduction.

**Figure 5:**
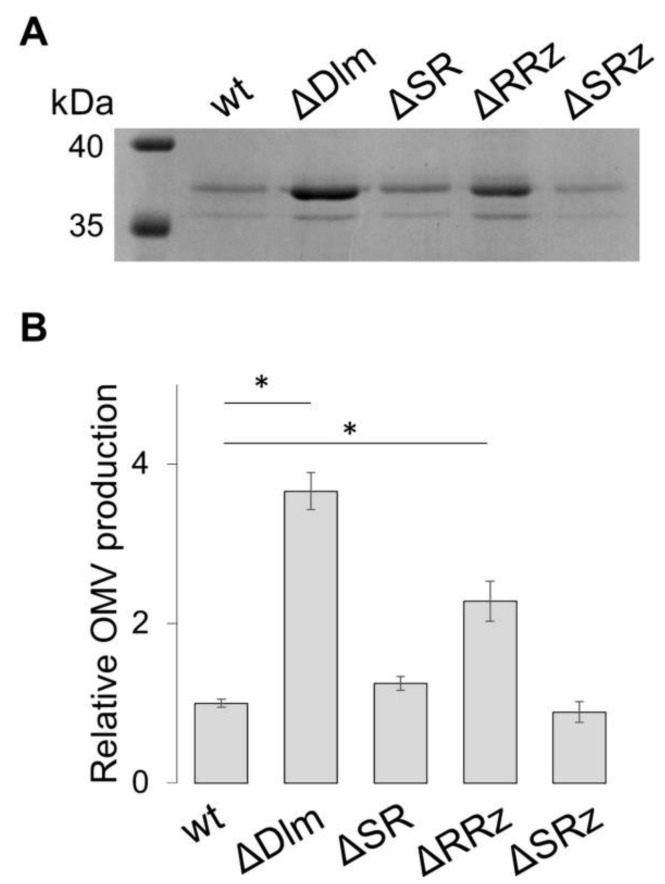
OMV production in ULS153 derivative strains lacking two genes of the DLP12 lysis module. (**A**) SDS-PAGE separation of OMV proteins from ULS153 (wt) and its derivatives lacking the entire DLP12 lytic module (ΔDlm), *essD* and *ybcS* (ΔSR), *ybcS* and *rzpD*/*rzoD* (ΔRRz) or *essD* and *rzpD*/*rzoD* (ΔSRz). (**B**) OMV production by ULS153 ΔDlm, ΔSR, ΔRRz and ΔSRz normalized to the wt. OMV quantification was obtained by densitometry of the OmpF/C band in SDS-PAGE separations. Vertical bars indicate the standard deviation of three independent experiments. Statistical significance was determined by the *t* test, and *p* values are as follows: * *p*  <  0.01.

Then we asked whether the simultaneous absence of more than one gene might promote a level of OMV production similar to the one observed in the ΔDlm background. To this end, we constructed ULS153 double mutants containing deletions of the *essD* and *ybcS* genes (ΔSR), or of the *ybcS* and *rzpD*/*rzoD* genes (ΔRRz), or of the *essD* and *rzpD*/*rzoD* genes (ΔSRz). As shown in Figure 5A in the two double mutants lacking *essD* (ΔSR and ΔSRz), OMV production attains a level closely comparable to the wt, whereas the silencing of both the *ybcS* and *rzpD/rzoD* genes (ΔRRz mutant) induces a clear increase (2.4-fold) in vesiculation (Figure 5B). This increase does not yet reach the same vesiculation level (i.e., 3.6-fold increase over the wt) produced by the absence of the entire SRR_z_/R_z1_ operon. All together these data suggest that, while the absence of both the endopeptidase and spannin activities favors vesiculation, while a strong increase of OMV production also requires the absence of holin.

### 3.3. Stress Factors Modulate the Expression of SRR_z_/R_z1_ Operon

**Figure 6:**
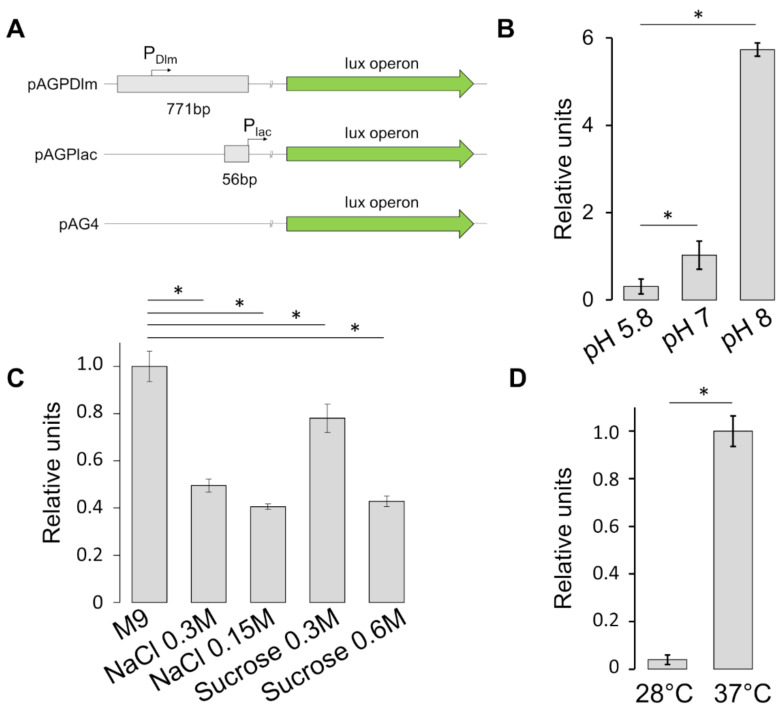
Expression of the DLP12 lysis module in response to stress conditions. (**A**) Schematic representation of pAGPDlm, pAGP*lac* and pAG4 constructs. PDlm activity as a function of pH (**B**), osmolarity (**C**) and temperature (**D**). Vertical bars indicate the standard deviation of three independent experiments. Statistical significance was determined by the *t* test, and *p* values are as follows: * *p*  <  0.01.

To gain more insight on the regulation of the SRR_z_/R_z1_ operon, we investigated whether environmental stimuli such as pH, osmolarity or temperature can modulate its transcription. To this end, a plasmid carrying a transcriptional reporter fusion, pAGPDlm, was constructed by cloning a fragment containing the regulatory region of the SRR_z_/R_z1_ operon (PDlm) just upstream of the promoterless *luxCDABE* operon carried by the pAG4 vector [33]. As a positive control, we constructed pAGP*lac,* a pAG4 derivative containing the *lac* regulatory region, without its operon box (P*lacO^-^*), upstream the *lux* genes (Figure 6A).

SRR_z_/R_z1_ expression as a function of pH was monitored by growing the ULS153 pAGPDlm, ULS153 pAGP*lac* and ULS153 pAG4 strains in M9 minimal medium at pH 5.8, 7, or 8. The luminescence levels were measured by means of the CLARIOstar plate reader (BMG LABTECH), and the results were calculated by subtracting the value of ULS153 pAG4 and comparing the resulting luminescence from ULS153 pAGDlm to that from ULS153 pAGP*lac*. As shown in Figure 6B, decreasing pH reduces Dlm expression. Next, to assay the influence of osmotic stress on the SRR_z_/R_z1_ promoter, we measured the luminescence emitted by ULS153 pAGPDlm, ULS153 pAGP*lac* or ULS153 pAG4 grown under different osmotic conditions. In order to distinguish between ionic and non-ionic osmotic stress [35,44] bacteria were grown in standard minimal medium (M9) or supplemented with either NaCl (0.15 M or 0.3 M) or sucrose (0.3 M or 0.6 M). The data (Figure 6C) indicate that SRR_z_/R_z1_ expression is repressed by both NaCl- and sucrose-mediated osmotic stress, though sucrose is less effective at the lower concentration tested (0.3 M). Finally, we monitored the effect of temperature on the same strains. The results of the luminescence assay (Figure 6D) reveal that SRR_z_/R_z1_ expression is repressed at 28 °C as compared to 37 °C. Altogether, these data strongly suggest that osmotic stress, low pH and low temperature negatively affect transcription of the lysis module.

## 4. Discussion

Phages play an important role in bacterial cell life and evolution. They contribute to several biochemical and physiological properties that increase the bacterial adaptation capability, including those involved in virulence, resistance to antibiotics and antimicrobial compounds, and biofilm formation [45,46]. In this context, temperate phages play a major role as once inside the host they can propagate vertically to successive cell generations (lysogeny). Co-evolution of the host and its prophage load often results in the decay of the phage genome and ultimately in the selection and preservation of prophage gene modules, i.e., sets of contiguous genes involved in similar functions and useful to face new environments and survive to stress [47,48]. This so-called *domestication* process is evidenced by the presence in bacterial genomes of several molecular systems encoded by prophage modules and having a beneficial impact on bacterial cell physiology [46,49].

In this context, DLP12 represents an interesting model. DLP12 is a *E. coli* K12 defective lambdoid prophage which has lost most functions related to the reactivation of the lytic cycle. Unexpectedly, its lysis module (Dlm) has been integrally conserved and is transcribed. Dlm consists of four genes grouped in a single operon, SRR_z_/R_z1_: the *essD* gene, coding for a holin/pinholin complex (S); the *ybcS* gene coding for an endolysin (R); and the *rzpD/rzoD* genes, two nested genes encoding spannins (R_z_/R_z1_). It has been proposed that the persistence of a functional SRR_z_/R_z1_ operon within the *E. coli* genome is related to bacterial cell wall maintenance [19]. During growth and division, a seamless organization of the cell wall is maintained, as the polymerization of new peptidoglycan is associated with a degradative process, resulting in the overall addition of new subunits [50]. Besides exploiting cellular autolysins, the degradation activity also depends on the holin/endolysin system of DLP12. In particular, the endolysin-mediated hydrolysis is thought to promote the degradation of non-recyclable peptidoglycan fragments, thus avoiding their potentially harmful accumulation in the periplasm [19].

Outer membrane vesicles (OMVs) are small spherical structures released from the outer membrane of Gram negative bacteria [3,4]. They carry periplasmic components within their lumen and are known to be involved in detoxification of bacterial cells. In particular, their production is increased when “residues” such as misfolded proteins, or fragments of LPS or PG, accumulate in the periplasmic space [13,14]. Given the role of the SRR_z_/R_z1_ operon in PG dynamics [17,19], we have investigated whether a relationship between this operon and vesiculation exists.

The results shown in Figure 1 indicate that the loss of the entire lytic module of DLP12 causes an about 4-fold increase in OMV production. This level is well comparable to that attained by other *E. coli* mutants, e.g., DegP, whose hypervesiculating phenotype has been widely studied [13,15,43]. Strains harboring mutations in PG hydrolases, such as endopeptidases or transglycosylases, also cause hypervesiculation [13,14]. This has been related to the fact that a decreased PG degradation may lead to an excess of cell wall material, which triggers OMV production. On this basis, it can be assumed that the loss of the DLP12 lysis module leads to an increase of non-recyclable PG fragments with consequent bloating of the periplasmic space. Also in this case the enhanced vesiculating response can be regarded as a means adopted by the bacterium to free-up the periplasm from undesirable material by delivering it to the outer environment.

What is the contribution of the individual genes of the SRR_z_/R_z1_ operon? The analysis of deletion mutants (Figure 4) indicates that the loss of single genes increases OMV production only slightly, i.e., from about 1.3-fold (ΔS mutant) to about 1.7-fold (ΔR and ΔRz). This strongly suggests that the entire operon has been domesticated by the host bacterium to increase the degradation efficiency of unrecyclable PG fragments. When double mutants were studied (Figure 5), we observed that the presence of holin (*essD* gene) in concomitance with the absence of corresponding endopeptidase and spannin (*ybcS* and *rzpD/rzoD* genes) is accompanied by an about 2.4-fold increase of vesiculation. As suggested by previous studies on phage λ [51], this may be due to holin favoring the transit of “residue/waste” molecules through the inner membrane towards the periplasmic space. Indeed, our data indicate that DLP12 single or double deletion mutants lacking holin (ΔS, ΔSR and ΔSRz) show vesiculation levels closely comparable to the wt (Figure 4 and Figure 5).

As the absence of a functional DLP12 lysis module increases OMV production, we asked whether stress conditions could decrease the expression of the SRR_z_/R_z1_ genes thus favoring vesiculation for a rapid elimination of potentially harmful residues. By means of a reporter fusion approach, we monitored the effect of temperature (28 °C vs. 37 °C), osmotic (ionic and no-ionic hyper-osmosis) and pH stress (pH from 5.8 to 8) on *essD* transcription. The results (Figure 6) indicate that the expression of the SRR_z_/R_z1_ operon is sensitive to all the tested conditions and that environmental stresses as low temperature, acidic pH and high osmolarity lead to repression. We can argue that OMV production and optimization of peptidoglycan recycling are sensitive to stressing conditions as these may, somehow, induce over-stretching of the cell envelope and/or other undesirable or poorly tolerated biochemical modifications.

The functional relevance of prophage lysis modules has also been observed in processes such as biofilm formation [19,52] and the secretion of virulence factors [26,53]. Vesiculation is known to be subjected to the control of several genes involved in the biosynthesis of the cell envelope [14]. The present study has allowed us to identify and characterize a new genetic element involved in the regulation of OMV production. While the molecular mechanisms of this process remain to be fully elucidated, the very fact that a prophage remnant as the DLP12 SRR_z_/R_z1_ operon has been domesticated to regulate OMV production is intriguing, as it adds a new layer of complexity to the dynamics of OMVs in bacterial cell life. In general, this finding further highlights that defective prophages cannot be considered as simple carriers of genetic baggage, since they can actively contribute to the host physiology and fitness [46,48,49].

## Supplementary Materials

The following are available online at https://www.mdpi.com/2076-2607/9/2/369/s1, Table S1: Oligos used in this study.
