# Supplementary material for: Modulation of OMV Production by the Lysis Module of the DLP12 Defective Prophage of Escherichia coli K12
Source: Microorganisms. 2021 Feb 12;9(2):369. doi: 10.3390/microorganisms9020369 (PMC7918800; doi:10.3390/microorganisms9020369)
Supplement: Supplementary file 1 [file microorganisms-09-00369-s001.pdf]

microorganisms

MDPI

Supplementary Materials: Modulation of OMV Production by the Lysis Module of the DLP12 Defective Prophage of *Escherichia coli K12.*

Pasqua M, Zennaro A, Trirocco R, Fanelli G, Micheli G, Grossi M, Colonna B and Prosseda G*

Table S1. Oligos used in thi study

Name	Sequence (5'-3')	Used for
**datDlm_F**	CCTCAGCAGGTAGTGCCGGGTACTGGTTTTTACAGCGTG-TAGGCTGGAGCTGCTTC	deletion of DLP12 operon
**datDlm_R**	GGCTTCACGCACTGACTGACAGACTGCTTTGATGTGAT-TCCGGGGATCCGTCGACC	deletion of DLP12 operon
**datS_F**	ATGGATAAGTTAACAACGGGTGTCGCCTATGGCACCTGTG-TAGGCTGGAGCTGCTTC	deletion of essD gene
**datS_R**	AGCAGCAGCAACGGCTTTTCGTAATGATGGAGGCCATATGAAT ATCCTCCTTA	deletion of essD gene
**datR_F**	CAATTGCTATAGCATCAGTGTTAATCACTGGCCCAATGTGTAGG CTGGAGCTGCTTC	deletion of ybcS gene
**datR_R**	TATAATCGCGGTTACTCTGCTCACTGTGCCCCCATATGAA-TATCCTCCTTA	deletion of ybcS gene
**datRz_F**	TCCGCTCTGATTATCTGCATCATCGTCAGCCTGTCATGTG-TAGGCTGGAGCTGCTTC	deletion of rzpD/rzoD genes
**datRz_R**	GTTGCCCATCGATATGGTCAGCTCTATCTGCAC-TGCCATATGAATATCCTCCTTA	deletion of rzpD/rzoD genes
**Pdlp12Fw**	NNNCTGCAGCAGCTGCCTGAGCAGACATC	DLP12 promoter region amplification
**Pdlp12Rv**	NNNCTGCAGCAGCACACCTATTGCTGCCCAC	DLP12 promoter region amplification
**PlacFw**	GATCTCATTAGGCACCCCAGGCTTTACACTTTATGCTTCCGGCT CGTATGTTGTGTGGA	Lac promoter amplification
**PlacRv**	GATCTCCACACAACATACGAGCCGGAAGCATAAAGTGTAAAGC CTGGGGTGCCTAATGA	Lac promoter amplification
